# How is the in-patient psychiatric ward round understood in research literature? Scoping review

**DOI:** 10.1192/bjb.2025.10139

**Published:** 2026-06

**Authors:** Benjamin Williams, Siobhan Richardson, Georgia Jameson, Oluwatomilola Olagunju

**Affiliations:** 1 Consultant Psychiatrist, North View, Greater Manchester Mental Health NHS Foundation Trust, Manchester, UK; 2 Advanced Clinical Practitioner, North View, Greater Manchester Mental Health NHS Foundation Trust, Manchester, UK; 3 Equality, Diversity and Inclusion Team, EDI Partner, Greater Manchester Mental Health NHS Foundation Trust, Manchester, UK; 4 Resident Doctor, Mersey and West Lancashire Teaching Hospitals NHS Trust, Prescot, UK

**Keywords:** Ward round, in-patient, general psychiatry, multidisciplinary team meeting

## Abstract

**Aims and method:**

This scoping review examines the literature on psychiatric in-patient ward rounds, a crucial and ubiquitous but understudied component of psychiatric care. We sought to examine the methods and perspectives used in research on ward rounds and identify recommendations for practice.

**Results:**

The review identified 26 studies from diverse in-patient settings but predominantly UK-based, which made 21 recommendations for practice. The commonest methods used were staff surveys and patient interviews. Patient experience, structure, efficiency and power dynamics were the commonest research foci.

**Clinical implications:**

Key recommendations for improving psychiatric ward rounds include reducing participant numbers, increasing patient involvement, structured documentation and regular scheduling. Despite weak empirical evidence supporting these suggestions, they are seen as feasible starting points for quality improvement. The review calls for future research to triangulate patient and staff reports with direct observation to better assess ward round effectiveness and outcomes.

Ward rounds are a long-held, essential, aspect of hospital tradition and the delivery of patient care.^1^ They are complex clinical activities that form a pivotal point of care designed to facilitate communication between doctors, nurses and members of the multidisciplinary team (MDT) to deliver good patient care and address their concerns.^2^ They may fulfil other roles such as training, safety checking and making discharge arrangements.^3,4^ Ward rounds may be held in several different formats. Regardless of the format of ward rounds and MDT composition, the role of the medical and nursing teams is always central to the process.^3^ They have practical and cultural importance as both tool and ritual in which bedside teaching occurs, and knowledge is passed down from senior doctors to their more junior colleagues.^5^ They remain a ubiquitous way to manage a team, facilitate a good relationship between the patient and doctor and pass news among the treatment team, the patients and their family members, in both medicine and psychiatry.^4^ In psychiatry, ward rounds fulfil many of the same functions as in general hospital medicine although usually occurring less frequently, often once or twice per week. Due to the complex environmental, social and clinical factors at play in psychiatry, there have been several difficulties associated with conventional ward round structure and the increasing numbers of professionals often present. These include issues with power dynamics between the consultant, staff and patients; difficulties in adequately communicating with patients that have altered mental status; and discussing intimate information with large numbers of healthcare professionals present.^6,7^ With a complex clinical intervention within a similarly complex environment, there is considerable variation in practice. There is no established gold standard in the UK as there is for internal medicine.

As with other aspects of in-patient psychiatry, ward rounds are often seen as coercive interventions within a predominantly biomedical approach.^8^ Practitioners in various disciplines have sought to reform or refine the practice. One notable area of inspiration for rethinking what constitutes successful patient–staff interactions has been therapeutic communities. Community meetings do not provide a direct analogy to ward rounds, their scope typically being much broader and meetings much more frequent. Nonetheless, therapeutic community ideals and principles have shaped the intellectual landscape through which good practice is viewed.^9^

For such a central part of everyday in-patient psychiatric practice, there has been a modest amount of research focused on psychiatric ward rounds. This scoping review aims to look at the methods used by perspectives researchers to understand ward rounds and to identify recommendations that could help inform our practice of ward rounds in psychiatry.

## Method

The review protocol was registered on the open science framework (link available on request).

The eligibility criteria were broad, to reflect the concern of the review. Studies were included if they featured discussion of psychiatric in-patient ward rounds in which patients were participants. Ward rounds did not have to be the primary focus of the research, but to constitute a key component of the research and have results described separately. For example, a paper on communication with patients that included both ward rounds and patient–nurse communication at other times, with clear distinction of when each is referred to, would be included. Both civil and forensic hospitals, or wards, were included. Acute psychiatric intensive care, specialist (for example, eating disorders or intellectual disability wards) and rehabilitation wards were included. No restrictions were made according to age, gender, diagnosis or nationality. No restriction was made for study design to consider various aspects of ward round functioning, and to allow triangulation between methods where possible. Studies were sought that addressed practical concerns, e.g. efficiency or comprehensiveness, or broader and theoretical concerns such as issues of culture, communication, group or individual psychology, power or identity as they occur during a psychiatric ward round. Included studies must be either peer-reviewed publications, research published as an editor’s letter or PhD theses.

Studies of professional discussions, handovers, board rounds or team meetings with no patient participation were not included. Studies of day hospitals and drug and alcohol rehabilitation services were excluded. Staff-facilitated community meetings or group psychotherapy in traditional in-patient settings or therapeutic communities were not included. Only English-language articles were included. Opinion pieces, editorials and editor’s letters not reporting research were not included.

The databases searched were Medline, CINAHL and ProQuest (including British Nursing Index, PsycInfo and Health Research Premium Collection). These were chosen to provide an overview of nursing, medical, psychological and allied health professional literature. The dates searched were from database inception to 13 November 2023. Reference checking was undertaken manually during the full-text screening process. Citation checking was undertaken for included studies using Crossref. Searches were limited to full-text, peer-reviewed papers and theses/dissertations. For ProQuest, search terms were limited to anywhere but full text. The search terms used were psychiatry* AND (ward round OR ‘multi-disciplinary team meeting’ OR ‘clinical team meeting’).

Search results were exported to Rayyan version 1.3.1 (Rayyan Systems Inc., Cambridge, MA, USA; https://www.rayyan.ai). Rayyan was used for algorithmic detection of duplicates, which were confirmed by manual checking with one author. Titles and abstracts were screened for inclusion by one team member using a broad approach to screen within articles. Three reviewers undertook full-text screening. All reviewers screened the same three first journal articles and discussed the results of these to provide calibration; all three reviewers then reviewed the remaining full texts independently. Differences in screening were resolved by a simple majority. The three reviewers undertook data extraction independently using the same chart. The same three papers as initially screened were also used for pilot data extraction, with minor changes and additional prompts added to the data extraction tool. Data charting was conducted using a spreadsheet. Data were initially extracted as wholesale as possible from the original papers. The authors then compared the data extracted and reduced these to common constituent parts or themes through discussion, with disagreements resolved by a simple majority.

The variables sought included each study’s definition of a ward round, theoretical basis, substantive focus, method for data collection and analysis, key findings, and recommendations for practice, as well as country, setting/speciality, participant numbers of staff and patients and participant demographics. Due to the heterogeneity of the study designs included, and the emphasis of the review on understanding the scope of the literature in a broad sense, no standardised tool for critical appraisal of the studies could be used. Assessment of the key strengths and weaknesses of each study was made independently by three authors contemporaneously with data extraction, and harmonised in the same fashion as the other data extraction.

Data synthesis was conducted by grouping studies according to theoretical concern and primary methodology. Recommendations for practice were harmonised and then presented, with the degree of support indicated by the number of studies in which they were recommended.

## Results

**Fig. 1:**
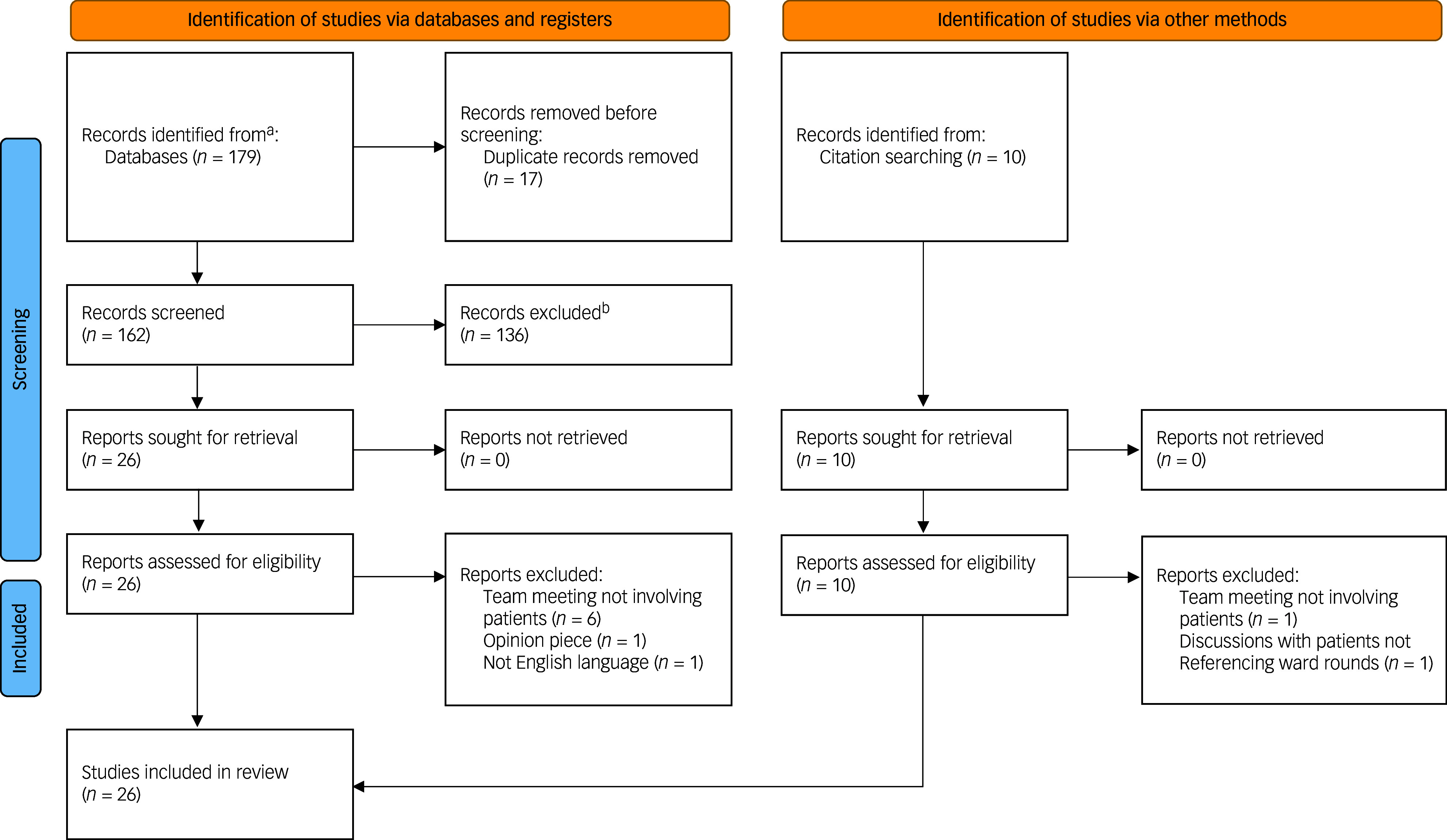
PRISMA 2020 flow diagram for new systematic reviews that included searches of databases, registers and other sources.

Twenty-six studies were included in this scoping review (Fig. 1); Table 1 presents the primary data extraction. Of the 26 studies, 21 were from the UK, 2 from Germany and 1 study was completed in each of Republic of Ireland, Australia and South Africa. The majority were set within general acute psychiatry settings, with a few in a range of other settings (low secure, psychiatric intensive care units, old age, eating disorder, rehabilitation, medical and surgical wards and perinatal units).

Of those studies using a method that recruited participants, the range of patient participants across 16 studies included was 5–301, with a median of 26 and a mode of 6, 8 or 10. The range of staff participants in the 13 studies included numbered 9–290, with a median of 31 and a mode of 21. The difference in participant numbers reflects more low-number, in-depth interview studies being carried out with patients and more survey studies of staff.

Not all studies offered a definition of an in-patient psychiatric ward round. Features of a definition included in more than one study were the following: being scheduled; being conducted away from the bedside; being consultant led; involving a MDT; having patient involvement; involving patient assessment; creating or reviewing a treatment plan and risk assessment; and being an opportunity for teaching.

The majority of studies did not use a specific theory. However, among those that used a specified theory, two used lean methodology, two used critical realism with one each using systems research, the phenomenological–hermeneutical method and constructionism.^10–16^ We did not find any studies informed from a specific psychological therapeutic perspective. One study, Rapsey et al, used a trauma-focused approach.^17^ While trauma-focused therapy has been defined as ‘any therapy … in which the trauma focus is the central component’ rather than as a traditional school of psychology, it still offers a different theoretical perspective.^18^ Despite this difference in focus, Rapsey et al obtained the same themes as those found by the other qualitative studies using interviews or focus groups.^17^ In those studies focused explicitly on power, and in some focused on experience in which power, control and hierarchy featured prominently, the influence of critical theories was evident although not explicitly elaborated upon in the manuscripts.^6,13,19–22^

The experience of ward rounds was the focus of nine studies,^7,17,20,21,23–27^ the structure of ward rounds was the focus of five and ^15,28–31^ efficiency was the focus of three.^10,11,32^ Power was the explicit focus of three papers,^6,13,19^ shared decision-making the focus of two^12,33^ and a unique focus was found in four.^14,16,34,35^

The most common methods used were cross-sectional surveys in 11, interviews in 11, audit or plan-do-study-act cycles in 3, time use survey in 2, ethnographic methods in 2, focus groups in 2 and personal reflection in 1. Several studies combined surveys and interviews. We identified no experimental studies or non-ethnographic longitudinal studies.

A small number of studies described innovations involving substantial changes to traditional ward round structure. Hodgson et al, on the other hand, found no viable alternative to the traditional ward round.^30^ Two studies examined group models of ward rounds;^31,36^ these concluded that such a change shifted the focus away from illness towards patients’ practical and social concerns. Fiddler and colleagues described the change process of moving from a weekly to a daily ward round model.^15^ That paper focused on the process of change rather than the effect of the changes themselves, concluding that it is possible to make changes to such an established practice as in-patient ward rounds.

O’Reilly and colleagues did not specify any recommendations for ward rounds, but posited that future research on these should consider mixed quantitative methods.^13^ They found a discrepancy between their observations of decision-making in ward rounds and patients’ reported satisfaction with these decisions outside of them. Wagstaff and Solts drew similar conclusions, although it was not the focus of their research.^25^ They observed that patients often reported not being able to recall being introduced to the professionals present at the ward round, when in fact they had been.^14^

From the findings of the studies, several recommendations were given. Many of the studies made recommendations for the conduct of ward rounds (Table 2). Recommendations made by more than one study included fewer people in ward rounds; increasing patient involvement; dedicated preparation time; providing feedback or access to ward round records; having structured ward round documentation; regular appointment times; focusing on preparing patients for discharge; and providing information about ward rounds. Each of the following were recommended by one study each: family involvement; peer advocacy; the availability of refreshments; the use of hybrid ward rounds (in both person- and technology-assisted ward rounds); considering the use of a group format for ward rounds; having the names of professionals involved in ward rounds listed on the door; having familiar staff present; ad hoc daily ward rounds rather than weekly and structured; addressing patients directly when using interpreters; pharmacist involvement in ward rounds; and, lastly, staff given instruction about how to do ward rounds, in addition to more teaching taking place during ward rounds.

**Table 1:** Summary of papers included in the scoping review

Study	Theory	Focus	Result
Cappleman et al^6^	None specified	Power	Themes: wanting more involvement in the process of ward round; the importance of relationships, power and control; considering patients’ emotional state.
Carey et al^21^	None specified	Experience	Themes: not knowing time, power and control; not knowing who will be there; provokes feelings of anxiety.
Chapman et al^27^	None specified	Experience	Overall satisfaction with ward round is high, but poor understanding of the purpose of ward round itself; anxiety linked to high numbers of attendees; and unclear purpose of involvement of non-medical members.
Coffey et al^14^	Systems research	Unique focus	Ward rounds can be positive or negative; ward rounds often feel unplanned; regular scheduling with a specific time slot generates anxiety in the build-up to ward round; more people present increases stress.
Curtis et al^10^	Lean methodology	Efficiency	Using a lean approach was effective; using a checklist improved the consistency of ward round task completion.
Fewtrell and Toms^31^	None specified	Structure	In groups, patients spoke increasingly about social problems than traditional ward rounds.
Fiddler et al^15^	Phenomenological–hermeneutical method	Structure	Themes: ‘bound by tradition’, ‘juggling the change’ and ‘light at the end of the tunnel’; many attendees being negative was also mentioned, although this is not a core theme.
Foster et al^26^	None specified	Experience	Most patients found the traditional ward round helpful, minority groups and men less likely to do so. Frequent concerns included being unclear on purpose of ward round, feeling anxious, feeling worried about confidentiality, having too many attendees, being unclear on timing and it being the only way to see the consultant.
Hodgson et al^30^	None specified	Structure	Median attendees of ward rounds, *n* = 7; SHOs mostly take notes and present histories; nursing and psychiatry always represented; variable MDT involvement; introductions consistently made; seating consciously arranged; pharmacy and psychology underrepresented.
Holzhüter et al^33^	None specified	SDM	Clear agenda correlated with being more involved in shared decision-making; what appears to observers to be paternalistic can be satisfactory for patients.
John^12^	Critical realism	SDM	Ward rounds present challenges to shared decision-making, often driven by institutional needs.
Kidd et al^20^	None specified	Experience	Mixed feelings from patients; patients appreciate discharge focus; staff value opportunity for MDT discussion; tension between MDT inclusion and rigidity; time pressure; mixed effect on patient–staff relationships.
Labib and Brownell^7^	None specified	Experience	Concerns around: waiting time; too many attendees; not feeling listened to; feeling anxious; feeling that information was being withheld. Meeting consultant before ward round was associated with better satisfaction.
Lennard^20^	Lean methodology	Efficiency	Dedicated ward round nurse improves communication and continuity.
Mattinson and Cheeseman^32^	None specified	Efficiency	Using a standardised template was generally positively received but may increase time demands; it had no impact on length of stay.
Milner et al^34^	None specified	Unique focus	A substantial minority of patients and staff do not understand the purpose or process of ward rounds; anxiety about ward rounds and too many attendees was common; pre-ward round preparation was found to be beneficial.
Noble and Billett^16^	Constructionism	Unique focus	Pharmacist involvement in ward rounds may build professional links with doctors and could make prescribing practices safer.
O’Reilly et al^13^	Critical realism	Power	Length of discussion regarding medication choices may not be associated with patients’ perception of being listened to; not agreeing may be confused with not listening; patients may not articulate objections highlighting the importance of advocacy.
Rapsey et al^17^	None specified	Experience	Themes: control and choice; lack of collaboration; negative emotional impact; stagnation; social discomfort; personalised care.
Roche et al^28^	None specified	Structure	Themes: timing; location; MDT involvement; ward round processes; patient experience; documentation.
Swartz^19^	None specified	Power	Ward rounds include transformation and socialisation of both staff and patients; power dynamics in ward rounds are multifaceted, that assumptions about race can lead to both mislabelling of cultural differences as disease and disease as cultural difference; holistic approaches to care can be seen as dodging responsibility to treat; ward rounds form a microcosm that both reproduces and subverts broader societal expectations and dialogues.
Turel et al^29^	None specified	Structure	Technology-assisted ward rounds substantially improve CPN attendance, although CPNs themselves mostly did not think that this improved patient care.
Vietz et al^35^	None specified	Unique focus	Shared competencies between surgical and psychiatric ward rounds were needed for (a) collaborative clinical reasoning, (b) clinician–patient communication, (c) clinician–team communication, (d) organisation, (e) teamwork, (f) management of difficult situations and error management, (g) self-management, (h) teaching, (i) empathy and (j) non-verbal communication. Less emphasis on clinical skills and more on empathy and non-verbal communication in psychiatry.
Wagstaff and Solts^25^	None specified	Experience	Themes of internal and external processes. Internal: satisfaction, negative feelings, feelings about consequences, coping with it. External: decision making, communication, number of people, practical arrangements.
White and Karim^24^	None specified	Experience	Patient preference for clear times and fewer than four people; no clear overall patient preference for ward round location. The majority found open discussion difficult in ward round.
Yim et al^23^	None specified	Experience	Ward rounds perceived as important but impersonal; anxiety was common, and staff and patients had divergent views regarding goals; patients felt uninvolved but were speaking for 48% of the time; mean 14.5 min duration of ward rounds.

MDT, multidisciplinary team; SHO, senior house officer; CPN, community psychiatric nurse; SDM, shared decision-making.

**Table 2:** Recommendations made by papers in the scoping review

Recommendation	No. of studies	Studies	Recommendations	No. of studies	Studies
Fewer people in ward rounds	8	Cappleman et al^6^, Carey et al^21^, Foster et al^26^, John^12^, Kidd et al^20^, Rapsey et al^17^, Wagstaff and Solts^25^, White and Karim^24^	Peer advocacy in ward rounds	1	John^12^
			Have refreshments available	1	Roche et al^28^
Increasing patient involvement	7	Cappleman et al^6^, Carey et al^21^, Coffey et al^15^, Fiddler et al^16^, John^12^, Wagstaff and Solts^25^, Holzhüter et al^33^	Ad hoc appointments daily	1	Fiddler et al^16^
			Address patients directly when using interpreters	1	Swartz^19^
Dedicated preparation time	7	Cappleman et al^6^, Chapman et al^27^, John^12^, Kidd et al^20^, Labib and Brownel^17^, Lennard^11^, Holzhüter et al^33^	Known staff in ward round	1	Roche et al^28^
			Consider a group format	1	Fewtrell and Toms^31^
Summary/feedback to be given	5	Curtis et al^10^, Chapman et al^27^, Coffey et al^15^, Roche et al^28^, Yim et al^23^	On ward door, list professionals present	1	Rapsey et al^17^
Checklists/agenda	5	Curtis et al^10^, Kidd et al^20^, Mattinson and Cheeseman^32^, Holzhüter et al^33^, Vietz et al^35^	More teaching within ward rounds	1	Vietz et al^35^
Regular appointment times	4	Labib and Brownell^17^, Roche et al^28^, Wagstaff and Solts^25^, White and Karim^24^	Hybrid ward rounds (both face to face and virtual)	1	Turel et al^29^
Patients should have access to ward round records	3	Cappleman et al^6^, Holzhüter et al^33^, John^12^	Routine pharmacist involvement	1	Noble and Billett^16^
Ward rounds should prepare patients for discharge	2	John^12^, Kidd et al^20^	Mixed quantitative methods help in studying ward rounds	1	O’Reilly et al^13^
Give patients information about ward rounds	2	Cappleman et al^6^, Milner et al^34^	Greater family involvement	1	White and Karim^24^

## Discussion

We have conducted, to the best of our knowledge, the first review of research literature focused on psychiatric ward rounds, and collated the recommendations for practice made in that research. Throughout the literature a key tension is apparent, although rarely directly addressed, between having too many people in the room and MDT working. Much of this seems to stem from the fact that ward rounds are routinely used as an opportunity to assess the patients’ mental state, which feels very exposed in a room full of often unfamiliar faces.^6,14,23^ However, this conflicts with patients’ wishes for greater involvement in their care, which relies upon all relevant decision-makers being present when decisions are made. In models of care in which the primary decision-making forum is MDT meetings with no patients present, these are largely excluded from those decisions. Moreover, non-medical professional groups can define their role in in-patient psychiatric care as encompassing advocacy for patients in medically orientated systems, which would be lost without the MDT.^37^ This results in trade-offs between overcrowding, collaboration and efficiency.

Of the few studies to have examined major changes to ward round structure in resolving such tensions, no studies were designed to show therapeutic or service level benefit. Both of the group round studies are over 35 years old, and it is unclear whether these models could be adapted to meet current institutional, privacy and other regulatory concerns.^31,36^ Fiddler et al’s change to daily ward rounds with more frequent, shorter and flexibly timed patient reviews goes against the recommendations made in most, but not all, other papers recommending regular appointment times.^14,15^ Moreover, Fiddler et al’s study was not designed to compare patient or staff satisfaction, but focused on the change process itself. The daily meeting approach in this study, although not explicitly cited, may draw from approaches used in therapeutic communities that typically have daily community meetings.^9^ One might consider alternative approaches to ward rounds within the wider existing model of in-patient working: for example, having weekly brief and focused practical MDT ward rounds by appointment with each patient, family and external professionals alongside individual informal medical reviews. This could reduce the level of exposure felt by patients in a room crowded with professionals, and would remove the concern that an unsuccessful ward round means another week wasted.^26^ Another approach could be that of Hansen and Slevin who, based on therapeutic community principles of reality confrontation and communalism, introduced twice daily community meetings alongside weekly ward rounds.^38^ Such suggestions are speculative and likely to have significant implications for staff time, unless strict boundaries around meeting times and the independent purpose of different meetings are maintained.

The recommendations identified aimed at improving ward rounds, in their current most common format, hold good face validity in the view of the authors. However, there are no experimental studies implementing these recommendations. As demonstrated by Hansen and Slevin’s implementation of therapeutic community principles, cluster-randomised trials of such changes are feasible.^38^ The three studies using audits, or plan-do-study-act cycles, did show that it is possible to improve the consistency of ward round content and documentation.^10,29,32^ Only one assessed the impact on length of stay, and did not find a difference.^32^ Overall, the studies identified either were not designed to implement their recommendations or had limited or no ability to assess changes in key outcomes such as patient and staff satisfaction, quality of intra-round communication, objective measurement of patient involvement or length of hospital stay. Thus, the empirical support for the recommendations from this review is weak. It is our view that the recommendations collated in this review are unlikely to cause harm. In collecting the current available evidence on psychiatric ward rounds, and in the absence of more robust evidence, our findings could be considered for use as a starting point for quality improvement projects.

The strength of the evidence is particularly important to consider when several of the most common recommendations have significant implications for clinical resources, principally staff time. This includes pre-ward round meetings, post-ward round debriefings, pharmacist involvement and greater family involvement. Staff time as a resource is a major limiting factor on, the ability to implement the recommendations in this review. In the UK, as in other countries, staffing vacancies and increasing administrative responsibilities being placed on front-line nursing, medical and allied health professionals create considerable pressure.^39^ Improving patient experiences of ward rounds is likely to involve some additional input of these limited resources.

Our review has highlighted the need for future research on ward rounds in vivo, to triangulate a combination of patient and staff reports with direct observation. Many of the included qualitative studies relied solely on patient reports with close alignment to constructivist approaches. This has led to neglect of consideration of external events and specific aspects of the clinical context. Quotes used in this body of research, such as patients describing feeling anxious because of ‘not hearing what I want to hear’, were almost entirely devoid of clinical context.^20^ There may or may not be extremely sound reasons, such as risk of suicide, why patients do not hear what they want to hear in ward rounds. Alternatively, the person may have a disorder that affects their interpersonal functioning and would be expected to influence their interpretation of ward round interactions. Perplexingly, the interview studies we identified gave minimal, if any, consideration to how the fact that their participants were suffering a mental disorder sufficiently severe to warrant hospital admission might influence the data obtained in interviews. In the three studies that did triangulate between interviews and observations, important discrepancies were found between patients’ perceptions and events as they occurred.^13,23,25^ The lack of triangulation with either direct observation or clinical information is a major limitation of the qualitative studies included in this review. Moreover, differences between patients’ memories of their ward rounds and what happened in those ward rounds are informative as a potential subject of future inquiry in itself. For example, one might wish to understand why patients may still report feeling uninvolved in decision-making despite being the main talkers in rounds, or why they are unable to recall being introduced to those present.^23,25^

Within the psychodynamic approach to research on therapeutic communities, attention is specifically drawn to such seeming contradictions.^9,40^ Contrastingly, we found an almost total absence of examination of interactions within ward rounds from any distinct psychological school. Because many of the study authors were psychologists or psychiatrists by profession, this was somewhat surprising. It seems to us that a variety of psychological lenses, from traditional psychodynamic to compassion-focused therapy, may hold valuable insights into understanding and improving communication within, and experience of, ward rounds for patients, families and professionals.

Similarly, there were no studies focused on the physical environment or sense of space. In fact, with a couple of exceptions, the only acknowledgement of physical space was alluded to via the sense of overcrowding or brief mentions of moving chairs.^17,19,30^ Perhaps this is because the ward round room is seen as either mundane compared with the revolutionary spaces such as the Paddington day hospital, or because studies of in-patient space have either focused on the in-patient setting broadly or on other particulars.^41^ Similarly, with the primary exception of Swartz, little attention was paid to the ritualistic nature of ward rounds.^5,19^ Roche et al did, however, recommend the provision of refreshments.^28^ Nevertheless, this was not put into the context of the near universal symbolism of hospitality indicated by such provision, or how this might be consciously employed to put all attendees on a more equal footing. Equally there was no discussion regarding how such provision might interact with broader clinical agendas regarding risk reduction if hot drinks are provided, or improving cardiovascular health if snacks are provided. Sharing refreshments between professionals and patients in ward rounds is one example of how the clinical ritual could potentially be changed, but such matters are not explored in the published journal literature.

As well as the limitations of the literature itself, our review has its own weaknesses. It was limited to those academic papers explicitly referencing psychiatric in-patient ward rounds in a searchable manner. There is undoubtedly a great deal of relevant material in other academic sources, books and the grey literature that provides accounts of in-patient admissions more broadly; these would not have been retrieved by our search terms. However, reviewing all literature on in-patient care for references to ward rounds would have been a prohibitively large task. Similarly, there is likely to be a large number of hospital audits and quality improvement projects not available to us. Most of the retrieved literature is from the UK, and we conducted our review in the English language only. It is unknown how much primary literature in other languages has not been included, or how the findings and recommendations from this review would translate to models of care in other countries.

In conclusion, we have conducted the first scoping review assessing the state of the published academic literature focusing on in-patient psychiatric ward rounds. We found that the majority of the published literature focused on psychiatric ward rounds used cross-sectional surveys or interviews. The experience of participating in ward rounds and matters of efficiency were the most common topics of research. Most of the identified literature did not have a clear theoretical underpinning. Future research on psychiatric in-patient ward rounds should consider triangulation in data collection, and focus on assessing the effectiveness of implementing changes. We have collated recommendations, with the frequency of those recommendations across papers providing some indication of the level of expert support for those recommendations.

## Supporting information

Williams et al. supplementary material Williams et al. supplementary material

## Data Availability

Data availability is not applicable to this article because no new data were created or analysed in this study.

## About the authors

**Benjamin Williams**, BM, BS, MSc, PhD, MRCPsych, Consultant Psychiatrist, North View, Greater Manchester Mental Health NHS Foundation Trust, Manchester, UK. **Siobhan Richardson**, BSc, Trainee Advanced Nurse Practitioner, North View, Greater Manchester Mental Health NHS Foundation Trust, Manchester, UK. **Georgia Jameson**, BSc, Equality Diversity and Inclusion Partner, Equality, Diversity and Inclusion Team, Greater Manchester Mental Health NHS Foundation Trust, Manchester, UK. **Oluwatomilola Olagunju**, MBBS, MClinEd, FHEA, MAcadMEd, Psychiatry Core Trainee 2, Mersey and West Lancashire Teaching Hospitals NHS Trust, Prescot, UK.
