# Supplementary material for: How is the in-patient psychiatric ward round understood in research literature? Scoping review
Source: BJPsych Bull. 2025 Sep 8;50(3):249–57. doi: 10.1192/bjb.2025.10139 (PMC13386320; doi:10.1192/bjb.2025.10139)
Supplement: Williams et al. supplementary material [file S2056469425101393sup001.docx]

| Study | country | speciality | *n* patients | *n* staff | Patient demographics | Staff demographics | Definition | Theory | Focus | Methodology | Key Findings |
| --- | --- | --- | --- | --- | --- | --- | --- | --- | --- | --- | --- |
| Cappleman 2015 | UK | General Acute | 5 | - | 1 female, age 20-49, LoS 1.5-11 weeks | - | MDT, Consultant chairing, Patient involvement, risk assessment, treatment plan | None specified | power | semi-structured interview with thematic analysis | Themes:  - wanting more involvements in the process,  - the importance of relationships  - power and control  - considering patients emotional state |
| Carey 2015 | Republic of Ireland | General Acute | 27 | - | 11 female, 70% voluntary, mean age 44, 41% LoS >4 weeks | - | MDT, Patient involvement, treatment plan, scheduled | None specified | experience | Cross sectional survey, descriptive and bivariate statistics | Themes:  - Not knowing when it will be,  - Power and control  - Not knowing who will be there  - Feelings of anxiety provoked by ward round |
| Chapman 2016 | UK | Forensic rehabilitation | 10 | - | 100% male, LoS 3-24 months | - | MDT, scheduled, patient assessment, teaching | None specified | experience | cross sectional mixed quantitative/qualitative survey with descriptive statistics and thematic analysis | -Overall satisfaction with ward rounds was high.  -Neither staff nor patients fully understood the purpose  - High numbers of attendees is anxiety provoking  - The purpose of the involvement of non-medical team members was unclear. |
| Coffey 2019 | UK | General Acute | 301 (36) | 290 (31) | - | - | None given | Systems Research | unique focus | cross sectional survey with ANCOVA and interviews with framework method | - Ward rounds can be positive or negative  - Ward rounds often feel unplanned - Participants disliked regular scheduling because having a specific time slot generates anxiety in the build-up  - High numbers of attendees was anxiety provoking. |
| Curtis 2014 | UK | PICU | 25 | 35 | - | - | MDT, patient assessment, scheduled, risk assessment, treatment plan | LEAN methodology | efficiency | PDSA cycle including cross sectional survey | - Using a lean approach was effective in improving the consistency of ward rounds.  - Using a checklist improved the consistency of ward round task completion. |
| Fewtrell 1985 | - | - | - | - | - | - | MDT, treatment plan, consultant chairing | None specified | structure | Time usage study comparing novel and traditional format | - In groups patients spoke more about social problems and less about symptoms than traditional ward rounds |
| Fiddler 2010 | UK | General Acute | - | 21 | - | 8 managers, 7 nurses, 3 psychiatrists, 2 OTs, 1 social worker; 5 to 30 years experience | MDT, scheduled, patient assessment, not at bedside | Phenomenological–hermeneutical method | structure | interviews with thematic analysis | Themes:  -‘bound by tradition’  -‘juggling the change’  -‘light at the end of the tunnel’  - The above themes describe the change process of adopting new ways of working.  - Having too many attendees having a negative impact was also mentioned though not a core theme |
| Foster 1991 | UK | General Acute | 50 | - | 38% female; age range 18-72; 72% white; 76% high school education | - | MDT, scheduled | None specified | experience | cross sectional survey using semi-structured interview analyses using descriptive/bivariate statistics | - Most patients found the with traditional wad round helpful  - Minority groups and men less likely to do so  - Frequent concerns included being unclear on the purpose, anxious, worried about confidentiality, too many attendees, being unclear on timing and ward rounds being the only way to see the consultant. |
| Hodgson 2005 | UK | General Acute | - | 96 | - | All consultant psychiatrists | MDT, teaching, patient involvement, patient assessment | None specified | structure | Cross sectional survey analysed using descriptive stats, plus free text | - Median attendees 7  - Junior doctors mostly take notes and present histories  - Nurses and psychiatrist always represented with variable involvement of other MDT members, with pharmacy and psychology under-represented.  - Introductions were consistently made.  - Seating consciously arranged. |
| Holzhüter 2021 | Germany | General Acute | 62 | - | 61% female; ages 18-78; 65% open wards | - | MDT, treatment plan, scheduled, teaching | None specified | SDM | Cross-sectional survey and observer ratings and with bivariate statistics | - Overall levels of shared decision making were assessed as low.  - Having a clear agenda correlated with more involved in shared decision making  - What appears to researchers to be paternalistic is often satisfactory for patients |
| John 2022 | UK | General Acute | 10 | 15 | 50% female; ages 30 to 80 | 73% female; 4 Occupational therapists, 3 nurses, 2 ward managers, 2 psychiatrists, 2 psychologists, 1 peer support worker, 1 healthcare assistant | Scheduled, Patient involvement, MDT, not at bedside | Critical realism | SDM | semi-structured interviews using framework analysis | - Ward rounds present challenges to shared decision making, these are often driven by institutional needs |
| Kidd 2023 | UK | Rehabilitation | 8 | 9 | 43% female; 86% white; ages 37-62; LoS 3-5 months | 56% female; 67% white; 3 nurses, 3 psychologists, 2 doctors, 1 OT. | Risk assessment, treatment plan, MDT, risk assessment, patient involvement, patient assessment | None specified | experience | semi-structured interview with thematic analysis | - Patients have mixed feelings regarding ward rounds  - Many patients appreciate being focussed on discharge planning  - Staff value opportunity for MDT discussion  - There are tensions between MDT inclusion and time pressures,  - The effects of ward rounds on patient-staff relationships was mixed |
| Labib 2009 | UK | General Acute and Perinatal | 42 | - | 31% female; ages 20-70; 57% detained | - | None given | None specified | experience | cross sectional survey, linear regression | - Patients had concerns around waiting time, too many attendees, not feeling listened to, feeling anxious, and a feeling information was being withheld.  - Meeting their consultant before the first ward round associated with better satisfaction. |
| Lennard 2014 | UK | General Acute | - | - | - | - | Scheduled, MDT | LEAN methodology | efficiency | personal reflection | - Dedicated ward round nurse improves communication and continuity |
| Mattinson 2018 | UK | General Acute and PICU | - | - | - | - | Scheduled, MDT, patient assessment, treatment plan | None specified | efficiency | PDSA cycle including cross sectional survey with descriptive statistics | - Using a standardised template was generally positively received by staff and improved consistency, but may increase time demands.  - It had no detectable impact on length of stay |
| Milner 2008 | UK | General Acute | 39 | 48 | 77% female; mean age 45; 18% detained; 54% LoS 1-4 weeks | 60% female; mean age 36; 22 nurses, 21 doctors, 4 social workers, 1 psychotherapist; 15% <1 year experience | None given | None specified | unique focus | Cross sectional survey, with descriptive stats and qualitative analysis | - A substantial minority of patients and staff do not understand the purpose or process of ward rounds  - Anxiety was common  - Too many attendees was a frequent concern  - Pre-ward round preparation is beneficial |
| Noble 2017 | Australia | Mixed including medical and surgical | - | 34 | - | 11 junior doctors, 10 consultants, 13 pharmacists | None given | Constructionism | unique focus | interviews with thematic analysis | - Pharmacist involvement in ward rounds may build professional links with doctors and could improve training and make prescribing practices safer |
| O'Reilly 2021 | UK | General Acute, PICU and Rehabilitation | - | - | - | - | Patient assessment, treatment plan, scheduled | Critical realism | power | Ethnography (combined observation and interviews), analysed with critical discourse analysis, | - Increased length of discussion regarding medication choices may not be associated with patients’ perception of being listened to  - Patients appeared to conflate not agreeing with them to not listening to them  - Patients may struggle articulate questions or objections within the ward round, highlighting the importance of advocacy |
| Rapsey 2023 | UK | Low secure unit | 6 | - | 100% male; ages 29-61 | - | Scheduled, MDT, patient involvement | None specified | experience | Focus groups and interviews with thematic analysis | Themes:  - Control and choice,  - Perceived lack of collaboration  - Negative emotional impact,  - Stagnation,  - Social discomfort  - Personalised care |
| Roche 2016 | UK | General Acute | 34 | 27 | ages 18-65 | - | MDT, scheduled, treatment plan, patient involvement | None specified | structure | semi-structured interviews with thematic analysis | Themes:  - Timing,  - Location  - MDT involvement  - Ward round processes,  - Patient experience  - Documentation |
| Swartz 1991 | South Africa | General Acute | - | - | all black south african women | predominantly white psychitrists and black nurses | MDT, patient assessment, treatment plan, teaching | None specified | power | ethnographic observation | Ward rounds include transformation and socialisation of both staff and patients  - Power dynamics in ward rounds are multifaceted  - Assumptions about race can lead to both mislabelling cultural differences as disease and disease as cultural difference  - Holistic approaches to care can be seen as dodging responsibility to treat  - Ward rounds form a microcosm which both reproduces and subverts broader societal expectations and dialogues. |
| Turel 2022 | UK | General Acute | - | 21 | - | 100% community psychiatric nurses | Scheduled, consultant chairing | None specified | structure | Retrospective audit with descriptive statistics | - Technology assisted ward rounds substantially improve CPN attendanc  - CPNs themselves mostly did not think this improved patient care |
| Vietz 2019 | Germany | Surgical, psychiatric and psychosomatic | - | 60 | - | Of psychiatric staff - 8 residents, 6 consultants, 8 nurses, and 8 psychologists; 47% female | MDT, patient involvement, treatment plan | None specified | unique focus | semi-structured interview with frequency analysis | Shared competencies between surgical and psychiatric ward rounds were:  - collaborative clinical reasoning  - clinician patient communication  - clinician - team communication  - organization  - teamwork  - management of difficult situations and error-management  - self-management  - teaching  - empathy  - nonverbal |
| Wagstaff 2003 | UK | General Acute | 8 | - | 63% female; ages 18-70; LoS 1-24 weeks | - | Treatment plan | None specified | experience | semi-structured interview with thematic analysis | Themes divided into internal and external processes.  Internal:  - satisfaction  - negative feelings  - feelings about consequences  - coping with it.  External:  - decision making  - communication  - number of people  - practical arrangements |
| White 2005 | UK | General Acute and Older Adult | 100 | - | 50% female | - | Scheduled, treatment plan | None specified | experience | cross sectional survey, descriptive statistics | Patient preference for clear appointment times and less than 4 people present in ward rounds.  No clear overall patient preference for ward round location.  The majority of patients found open discussion difficult in ward round. |
| Yim 2023 | UK | Adult Eating Disorders Unit | 6 | 10 | 100% female; 83% voluntary | 4 doctors, 3 nurses, 1 dietician, 1 advocate, 1 student | None given | None specified | experience | Time use survey, focus groups and interviews with thematic analysis and direct observations with behavioural analysis | Ward rounds perceived as important but impersonal, anxiety was common and staff and patients had divergent views regarding goals. Patients feel uninvolved but were speaking for 48% of the time. Mean 14.5 minutes duration. |

UK, United Kingdom; LoS, Length of Stay; MDT, multi-disciplinary team; CPN, community psychiatric nurse.
